# Health care providers’ responses to sexually abused children and adolescents: a systematic review

**DOI:** 10.1186/s12913-022-07814-9

**Published:** 2022-04-04

**Authors:** Mona Rahnavardi, Shadab Shahali, Ali Montazeri, Fazlollah Ahmadi

**Affiliations:** 1 Department of Reproductive Health and Midwifery, Faculty of Medical Sciences, Tarbiat Modares University, Tehran, Iran; 2 Health Metrics Research Center, Institute for Health Sciences Research, ACECR, Tehran, Iran; 3 Faculty of Humanity Sciences, University of Science &Culture, Tehran, Iran; 4 Department of Nursing, Faculty of Medical Sciences, Tarbiat Modares University, Tehran, Iran

**Keywords:** Child sexual abuse, Sexual violence, Adolescence, Systematic review, Child, Sexual abuse, Reproductive health

## Abstract

**Background:**

Sexual abuse of children and adolescents is a significant health concern worldwide. Appropriate and timely health services for victims can prevent severe and long-term consequences. This study identified and categorized diagnostic and treatment services needed for sexually abused children and adolescents.

**Methods:**

Several databases, including MEDLINE, Web of Science, Scopus, Science Direct, ProQuest, and Google Scholar, were searched to retrieve studies on the topic and clinical guidelines in English covering the literature from 2010 to 2020 using search terms. Primary studies and guidelines were reviewed to identify treatment strategies and medical interventions related to sexually abused children and adolescents.

**Results:**

Twenty-one studies and guidelines were selected and analyzed narratively. The quality of evidence was relatively good. We identified that effective health care systems for sexually abused children include the following: interview and obtain medical history, physical and anogenital examination, collecting forensic and DNA evidence, documenting all the findings, prevention and termination of pregnancy, diagnostic tests, prophylaxis for HIV and other STIs, vaccinations, and psychological intervention.

**Conclusions:**

This review provides up-to-date evidence about adequate health care services for children and adolescent victims of sexual abuse. We conclude that recent studies have focused more on prophylaxis against HIV and other STIs, studies on vaccinating against HPV for victims are still limited, and future research in this area is needed.

**Supplementary Information:**

The online version contains supplementary material available at 10.1186/s12913-022-07814-9.

## Background

Child Sexual Abuse (CSA) is a pervasive concern for public health and human rights [1]. The World Health Organization (WHO) has defined sexual abuse against children and adolescents as “involving a child in a sexual activity that he or she does not fully understand and has no informed consent because of the child’s intellectual development and immaturity.” Either it is an act that is not clear to the child or a violation of society’s laws or social restrictions [2]. The prevalence of child and adolescent sexual abuse is alarming. Three meta-analyses have been conducted to determine world-wide prevalence rates. A systematic review and meta-analysis in 2011 reported that the prevalence of CSA among girls to be 20% and among boys at around 8% [3]. Another 2013 meta-analysis about the prevalence of CSA reported that 9% of girls and 3% of boys had experienced forced oral, vaginal, or anal intercourse [4]. Meta-analyses by Stoltenberg et al. in 2015 reported that 8% of boys and approximately 18% of girls during their childhood had experienced sexual abuse [5]. Studies on children and adolescents exposed to sexual abuse confirm wide ranges of short- and long-term destructive health consequences [6, 7]. Sexual abuse can affect children and adolescents’ physical, sexual, reproductive, and mental health and need to be addressed [2]. Survivors of CSA may experience significant lifetime consequences in mental health, including depression, post-traumatic stress, anxiety, eating disorders, externalizing symptoms, problems with relationships, sleep disorders, and suicidal ideation and behaviors [7–9]. Studies have reported that children and adolescents who have been sexually abused are more likely to engage in high-risk behaviors such as risky sexual behaviors and drug and alcohol abuse, leading to adverse health consequences in adulthood [10–12]. Many survivors of sexual abuse can overcome such adverse health conditions as long as support services are provided and the confidentiality of their information and treatments insured [13–15].

Health care providers play an essential role in early access to services and care for child and adolescent victims of sexual abuse [16]. These victims need effective and immediate medical, psychological and legal services [17]. According to the World Health Organization, the rate of abused children presenting to medical and legal centers varies from country to country. For example, the rate of children with sexual abuse seeking help from health or legal centers in Swaziland was 24%, while in Kenya, it was only 2% for boys and 6.8% for girls [2]. These data indicate that there is a need to increase awareness in communities about the diagnosis and treatment options and provide timely services to victims of sexual abuse [15].

Although a recent systematic review by the World Health Organization (2017) explored the literature on good practice considerations for initial health system response to child and adolescent victims of sexual abuse [18], it only focused on how the provision of care to survivors should be implemented. In addition, this review did not synthesize the evidence on diagnostic and treatment services. Also, WHO 2017 guidelines need to be updated, as it does not include Tetanus vaccinations for high-risk children, laboratory tests for Hepatitis C, or the Papanicolaou test (Pap smear) for adolescent girls. Thus, the purpose of this review was to identify and categorize diagnostic and treatment services that are needed for sexually abused children and adolescents.

## Methods

### Design

This was a systematic review of the literature. We followed the Preferred Reporting Items for Systematic reviews and Meta-Analyses (PRISMA) guidelines.

### Search strategy

Six electronic databases, including MEDLINE; Web of Science; Scopus; Science Direct; ProQuest, and Google Scholar, were searched. We also searched databases such as Guidelines International Network (GIN), Agency for Healthcare Research and Quality (AHRQ), National Institute for Clinical Excellence (NICE), Unicef Guidelines, and World Health Organization (WHO), to retrieve clinical guidelines related to the research. Then, the keyword was adjusted based on the concept in the research question. These concepts consist of “Children” OR “child” OR “Minors” AND “Adolescent” OR “Adolescents” OR “Teenagers” AND “Sexual Abuse” OR “Sexual Assault” OR “Sex Offenses” OR “Rape” OR “Child Molestation” OR “Sexual Molestation” AND “Guideline” OR “Practice guideline” OR “Intervention” OR “Program” AND “Medico-legal assessment” and “Medico-legal intervention.” The final keywords were reviewed by a public health expert and two reproductive health experts. Studies published in English languages between 2010 and 2020 were included. The citations were transferred to the Mendeley Reference Management Software.

### Inclusion/exclusion criteria

Articles, guidelines and clinical and treatment protocols were included in this review if they met the following criteria: (1) Studies on sexually abused children and adolescents under 18 years of age of both sexes (boys, girls); (2) Studies that were conducted to identify treatment strategies and medical interventions related to sexually abused children and adolescents; (3) Studies on the legal interventions for victims of sexual abuse; or (4) Studies focusing on the rehabilitation and treatment of long-term consequences of sexual abuse.

Exclusion criteria for studies consisted of: (1) Studies on the prevention and protection of children from sexual abuse; (2) Studies on the characteristics and epidemiological distribution of sexual abuse in children and adolescents; (3) Resources on adults who have experienced sexual abuse in their childhood; (4) Studies focusing on other forms of child and adolescent abuse, such as physical or psychological violence; (5) Studies on the treatment and characteristics of sexual abuse perpetrators; (6) meta-analyses, and systematic reviews; and (7) Studies with transgender participants, disabilities, and other underlying diseases.

### Study selection process

The studies were classified, and then the full text of selected articles from the initial screening stage was searched and reviewed again by two authors. According to the inclusion and exclusion criteria, the first two authors conducted a primary title and abstract screening individually. Two reviewers (MR and SS) made separate judgments about the searched studies, and in case of disagreement, the third author (AM) made the final decision. Articles with study inclusion criteria were maintained for the quality assessment and data extraction stage.

### Quality assessment and risk of Bias

Studies were evaluated for scientific quality using the Mixed Methods Appraisal Tool (MMAT) version 2018 and its associated scoring system [19]. In brief, studies were scored based on five criteria as indicated by the guideline. Studies were rated as low quality if they met only 2 criteria and were excluded from the review. The remaining studies were rated medium if they met 3 criteria and high if they met 4 or 5 criteria. These studies were included in the review. In addition, the selected treatment guidelines for sexually abused children and adolescents also were assessed with the Appraisal of Guidelines, Research and Evaluation II (AGREE II) instrument [20]. The instrument contains 23 items divided into six sections. Scores on each section are independent. Guidelines with low quality (low scores on all sections or 4 out of six sections) were excluded. The remaining guidelines were rated medium if fulfilled adequate scores for 3 out of 6 sections, and high if fulfilled adequate scores for 4 or more sections.

### Data extraction process and data synthesis

The data extracted consists of: (1) general characteristics, (2) article summary, and (3) results of studies related to this review questions. General characteristics consisted of participant (Child or/and Adolescent), participant’s age and sex, and study location. The article summary included objective, research question, the methodology of studies (qualitative, quantitative; sample size), and main findings. The results included physical, psychological, forensic assessment and intervention, and follow-up services.

The studies included in this review were diverse in design, interventions, and primary and secondary outcome measures. As a result, meta-analysis was not applicable. The results were synthesized narratively as the best procedure for describing, comparing, and combining study findings.

## Results

### Search results

**Fig. 1:**
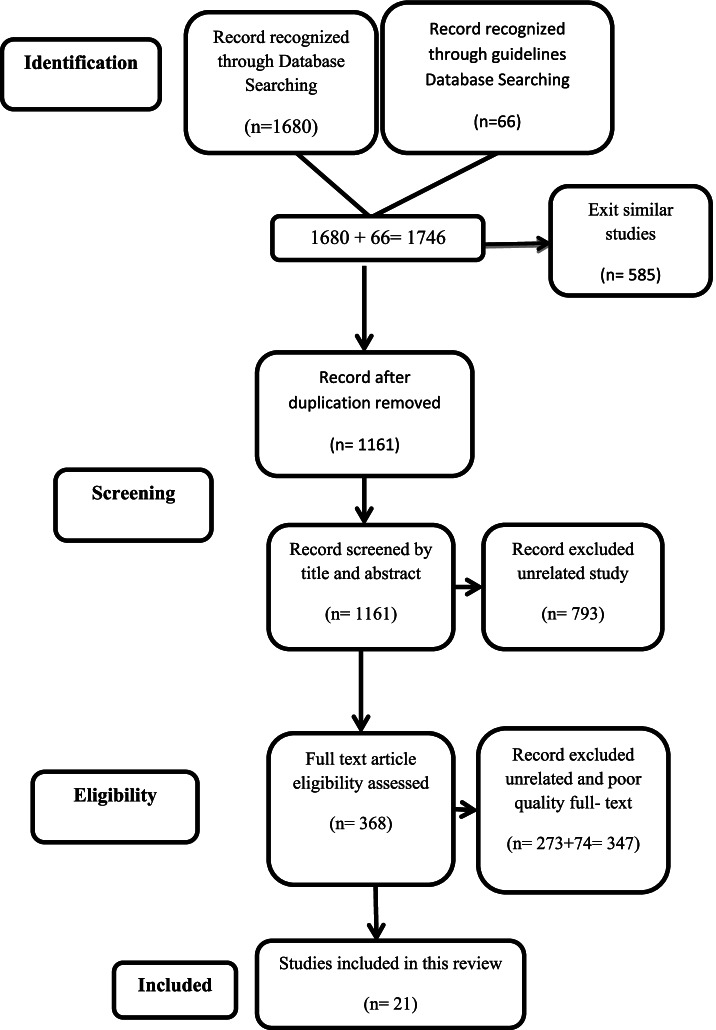
PRISMA diagram for Process of articles selection

The PRISMA flow diagram (Fig. 1) shows that the preliminary search yielded 1680 articles and 66 protocols or guidelines that potentially met the inclusion criteria. After excluding duplicates, 1161 articles and protocols were reviewed for the title and abstract. Consequently, 793 articles and protocols were excluded from the study. Then, the full text of 368 articles and protocols were assessed for eligibility. Out of 368 studies, 273 studies were excluded for the following reasons: focused on the epidemiological features of the child sexual abuse (*n* = 88), legal and medical measures against perpetrators of sexual abuse were examined (*n* = 71), the age of the sample was over 18 years (*n* = 43), assessed the prevalence and consequences of sexual abuse (*n* = 59), and the full text was not available (*n* = 12). In addition, 74 articles were excluded due to low quality. Finally, 21 articles and protocols were included in the review.

### Characteristics of studies under review

Out of twenty-one studies, 11 studies were clinical protocols for the management of child and adolescent sexual abuse [2, 21–30] (Table 1), 8 were quantitative [31–38], one was qualitative [39], and one article described a quality improvement project [40] (Table 2). The majority of the studies were conducted in high-income countries. Most quantitative studies were retrospectively conducted on sexually abused children and adolescents. Protocols or guidelines were developed by experts in child sexual abuse. One quantitative study was performed prospectively on 5 children with large anogenital ruptures [34]. The ages of children and adolescents in most studies were under 18 or 21 years old [2, 25, 26, 31, 32, 35–40], but in some studies, only the words “child” or “adolescent” were used, and exact age was not mentioned [23–25, 28, 30]. Two studies mentioned the combined care of adolescents and adults [21]. Data collection in two studies was in-depth interviews with children and their parents [37, 39]. Although most contents included all medical, psychological, forensic, and legal services for victims of sexual abuse, some addressed only one of the psychological [37], forensic [33], or HPV (Human Papilloma Virus) vaccination measures [36]. The quality of evidence was relatively good as can be seen in Tables 1 and 2. Overall, we identified seven services provided for children and adolescent victims of sexual abuse that were addressed by the studies under review. The findings are briefly described as follows.

**Table 1:** Characteristics of quantitative and quantitative studies included in this review

year	Author [ref.]	location	Time of Data Collection	methodology	Age and number of participants	Method of data collection	Quality Assessment	
							medium	high
2017	Harrison et al. [31]	LMIC (Zimbabwe)	September 2011 and December 2014	Quantitative	Adolescence (12-15 years) and adult (*n* = 3617)	Retrospective programmatic data collection		^a^
2012	Hornor et al. [32]	middle -Income (Ohio)	January 2004 to December 2007	Quantitative	1–20 years (*n* = 336)	Retrospective data collection of medical and legal records	^a^	
2011	Thackeray et al. [33]	middle-Income (Ohio)	January 2004 to December 2007	Quantitative	0–20 years	retrospective review of medical and legal records of patients	^a^	
2013	Sham et al. [34]	LMIC (India)	January 2009 to December 2010 with	Quantitative	4–11 years (*n* = 5)	prospective study analyzing case details and operative findings	^a^	
2015	Schilling et al. [35]	High- income (Philadelphia)	2004 to 2013	Quantitative	12–18 years (*n* = 12,687)	Pediatric Hospital Information System database		^a^
2018	Deutsch et al. [36]	High- income (Indiana, U.S)	October 2015 to October, 2017	Quantitative	Under 21 years (*n* = 448)	forensic nursing team database and patient electronic medical records		^a^
2018	Van duin et al. [37]	High- income (Amsterdam)	–	Quantitative	3–11 years (*n* = 44) And parents (*n* = 41)	police records and face-to-face interview		^a^
2018	Zerbo et al. [38]	High- income (Palermo)	October 2006 to December 2016	Quantitative	Under 16 (*n* = 90)	victims of sexual assault were retrospectively investigated		^a^
2016	Wangamati et al. [39]	LMIC^*^ (Western Kenya)	January 2013 to June 2014	Qualitative	14 years (*n* = 2)	Depth interview		^a^
2019	Goodman et al. [40]	East Carolina	–	Quality improvement project	9 years and older (*n* = 111)	Clinic records		^a^

^a^Low and middle-income country

**Table 2:** Characteristics of clinical guidelines or protocols included in this review

year	Author [ref.]	location	target group	Guideline development method	Intervention	Quality Assessment	
						medium	high
2018	B Ludes et al. [21]	ECLM^a^	victims of sexual violence	Not mention	all aspects of the medical and forensic examination		*
2016	Adams et al. [23]	North American Society for Pediatric and Adolescent Gynecology	Children	A group of specialists by review published research 2011 to 2014	Medical Assessment and Care		*
2013	Cybulska et al. [24]	UK^b^	victims of sexual violence	Not mention	Immediate medical care		*
2013	Jenny et al. [25]	AAP^c^	Children	Clinical report for updates an American Academy of Pediatrics	Primary care for child sexual abuse		*
2011	Cindy et al. [26]	Pennsylvania (USA)	Children	Not mention	Timing of the medical examination		*
2017	Crawford-Jakubiak et al. [27]	AAP	adolescent	clinical report for care acute sexual assault	acute sexual assault follow up and prevention		*
2017	KwaZulu-Natal health services [28]	KwaZulu-Natal health services	Children	Not mention	Medical examination and management	*	
2016	U.S. Department of Justice [29]	IAFN^d^ and OVW^e^	Pediatric	advisory from committee, as well as several organizations, associations, and individual	Medical and Forensic Examinations Pediatric		*
2018	Vrolijk-Bosschaart [30]	Amsterdam	Children	Clinical practice	recognizing child sexual abuse		*
2017	Moreno et al. [2]	World health organization	Child and adolescent	criteria and requirements stated in the WHO handbook for guideline development	Medical, psychological and forensic care		*
2013	Day and Weeks [22]	(PEPFAR^f^)	Child and adolescent	current, evidence-based practices	Clinical Management		*

^a^European Council of Legal Medicine

^b^United Kingdom

^c^American Academy of Pediatrics

^d^International Associations of Forensic Nurses

^e^Offices on Violence against Women

^f^United States President’s Emergency Plan for AIDS Relief

#### Principles for interviewing and obtaining medical history

General principles for interviewing children and adolescents who have been sexually abused include the following: use interviewers who have been trained in forensic medicine for forensic disclosure [2, 21, 23, 25, 27–30], build rapport with victims [2, 21, 22, 24, 26, 28, 29, 31, 38, 39], obtain informed consent from victims and their parents (except in cases where the parents are abusive), the examiner should be aware that any injured children may have been sexually abused, so a thorough examination of the relevant evidence is necessary for protect these victims [2, 21, 24, 26, 27, 34, 39], provide sufficient and clear explanations regarding all stages of the examination [2, 21, 23, 24, 26, 28, 30], provide a private and child-friendly place for interviews and ensure the safety of victims [2, 23, 24, 27, 28], preserve victims’ autonomy and avoid coercion [2, 21, 24, 25, 29], provide non-judgmental responses and support [2, 22, 25, 27], assurance regarding the confidentiality of information [2, 24, 39], use the child’s and adolescent’s language and give age-appropriate information [2, 24, 28], conduct interviews alone with victims [2, 23, 24, 39], use open-ended or general questions about themselves and events before the legal interview [2, 25, 27, 29, 39], record and document evidence [2, 21, 25, 29, 35], and ask for past medical and social history of all victims [2, 21, 22, 25]. The purpose of obtaining the medical history of victims is to check their health status, record the injuries, and ultimately address their health problems [2, 22, 24]. Also, to achieve the best treatment for children and adolescents, the examiner should attempt to obtain victims’ past medical records [2, 24, 25, 27] since such information may affect subsequent interventions, for example, victims who have previously received mental health interventions maybe need immediate psychiatric care [2, 27] .

#### Recommendations for physical and Anogenital examinations

Findings from 15 studies confirmed that when sexual abuse is discovered, the examiner should perform a complete physical examination to document any injuries. This examination is the first step in identifying and treating the injuries [2, 21–26, 28, 29, 31, 34, 38, 39]. it is difficult for the examiner to decide which child needs immediate sexual abuse examination and which case needs a referral to legal centers for more specialized examinations [25]. studies confirmed the conduct ofcomprehensive physical examinations and to avoid delay of examinations of the victims [2, 23, 25, 26, 28, 34, 35, 39]. Medical interventions to prevent complications in victims of sexual abuse depends on the early referral of sexual victims [26, 40]. Few studies have talked about victims’ fears and anxiety about physical and forensic examinations and have suggested that victims should have a sense of control and choice in examinations [2, 27–29, 39]. Four studies indicate that the presence of a supportive and empathetic caregiver or supportive person in the examination room can be useful for the child’s comfort [2, 25, 28, 29].

##### Physical examination

Studies show that in cases of child sexual abuse, the physical examination does not end only in the anogenital area, but a complete and comprehensive examination should be performed of all body parts [2, 21, 22, 28, 30]. Health care providers conduct a head-to-toe examination in the first stage of care [28–30]. Studies confirm that it is better to determine the stage of sexual maturity according to the Tanner stages. Then an anogenital examination is performed based on the stage of development [2, 21, 22, 28, 29]. Four clinical guidelines have recommended that every injury in all the skin surfaces, consisting of the soles of the legs, behind the ears, the eyes, the axilla, and the oral cavity, must be evaluated [2, 22, 28, 29]. If the victim’s interview reveals that oral penetration has also occurred, the victim’s oral cavity should also be sampled with swabs, and the swabs should be kept for microscopic investigation [21, 27, 28]. Any injuries to the victim’s body should be recorded [2, 22, 28, 29].

##### Genital and anal examination

To perform genital and anal examinations, visual aids can be useful for explaining the steps and position of the examination [2, 29]. Studies recommend that speculum, anoscope, and colposcope are not used to examine prepubescent girls. If the injury is severe and under anesthesia or sedation, then these instruments can be used [2, 24, 27–30, 34]. However, two studies indicate that a speculum can be used for intra-vaginal investigation [2, 25]. Most of the mentioned studies have suggested both supine (frog leg) and the knee-chest positions are better for genital and anal examination in both genders [2, 24, 27–30, 34, 39]. The genitals examination included examining the penis and scrotum in boys, and the labia and contents of the vestibule of girls, then documenting any injuries or scarring and other defects [25, 29]. Examiner should be checking any signs of injury or disease in victims and recognize whether this finding is normal or not [24, 29, 34] Then, first aid should treat minor injuries and, if necessary, refer to the hospital setting [24].

##### Collecting forensic and DNA evidence for forensic evaluation

Three studies confirmed that the time between the last event and evaluation is critical [26–28]. Most studies have stated that the best time to collect samples and identify the perpetrator is less than 72 h after the rape or sexual abuse [21, 25, 27–29, 35]. However, two studies from the USA reported that the time for the gathering of beneficial forensic evidence might be prolonged beyond the present 72-h standards if DNA testing is used for analyzing forensic specimens [26, 27] Samples, including blood, hair, semen fluid, and other likely foreign material can be obtained from the victim’s clothing and body skin, genitals, anus, and mouth by using swabs [21, 24, 27, 28].

##### Documenting findings from medical history and physical and forensic investigation

After observation and examination of victims, the examiner should completely and correctly document medical history findings, physical and forensic examination, and any other relevant evidence [2, 22, 24, 27–30]. Studies have even suggested that any examinations and injuries can be recorded with photo and videography [21, 22, 24–30, 34]. As a component of the informed consent process during the examination, consent to take video photography should be required [2, 29]. A national protocol for treating sexual abuse in children and adolescents recommended that video or audio recording during the medical history process not be used [29]; however, the WHO Clinical Guide recommended recording verbatim statements for perfect and comprehensive documentation [2]. The recorded documents must be securely stored in a safe setting [2, 29, 30].

#### Prevention and termination of pregnancy in sexually abused adolescent girls

Adolescent girls who have been sexually abused are at risk for unwanted pregnancies if they have attained menarche or are of reproductive age [2, 21, 22, 39]. Two studies report that pregnancy can signify sexual abuse in adolescent girls [23, 30]. Prevention of pregnancy can include emergency contraception for prophylaxis and termination of pregnancy [24]. Some studies state that after the contact sexual abuse in post-pubertal girls, emergency contraception for pregnancy should be given as soon as possible, preferably within 72 h after sexual abuse [21, 23–26, 28]. Some of the mentioned studies considered 120 h or 5 days after sexual abuse [2, 22, 25, 26, 35]. The initial response varied based on how much time has passed since the contact sexual abuse occurred [25]. One study in 2011 suggested that hormonal contraception is the only selective approach for pregnancy prevention in adolescent victims of acute sexual abuse [26]. But in WHO’s 2017 clinical guidelines for response to child and adolescent sexual abuse, intra-uterine devices such as copper-bearing intra-uterine device (Cu-IUD) are allowed if there is a low risk of STI [2].

Based on the recommendations of three clinical guidelines about abortion for pregnant adolescents, that decision should be based upon the local laws of each country [2, 22, 28].

#### Diagnostic tests

Children and adolescents who are victims of sexual abuse may need diagnostic tests based on their evidence and physical examinations. Based on the review of studies, these tests consist of the following:

- HIV (Human Immunodeficiency Virus): Since the frequency of HIV in prepubescent children and adolescents who have been sexually abused varies, diagnosis of these infections is recommended by almost all of the literature [2, 22–28, 30, 31, 35, 39]. However, two studies in the USA and UK suggest syphilis and HIV analysis should only be done in high prevalence populations or when the victims requested these tests [24, 35]. According to the result of a study in the USA, testing for HIV and other STI depended on the patient’s age, history of the abuse, timing of the medical examination, and perpetrator’s characteristics [26]. If warranted, HIV rapid testing (definitive results within 2 h) should be done. If the initial test results for HIV were negative, victims should repeat the test at 6, 12, and 24 weeks after the abuse [22, 28, 29].
- STI (Sexual Transmitted Infections): Studies confirm that identification of STI in pre-pubertal children can help to diagnose child sexual abuse, because it can be one of the possible signs of concealed sexual abuse [22, 28–30, 35]. Based on the review of studies, the most common STIs reported in victims of sexual abuse consist of chlamydia, gonorrhea, and trichomoniasis [22, 23, 26, 29, 30]. In this regard, some studies recommend vaginal culture in girls [26, 29, 35] or obtaining “dirty” (non-sterile) urine sample to detect Trichomoniasis [22]. Also, nucleic acid amplification test can be used to diagnose chlamydia, gonorrhea, and Trichomonas on vaginal swabs or urine samples during the first 72 h after abuse [2, 26, 28–30]. Furthermore, Chlamydia, Gonorrhea, syphilis, human papillomavirus, and herpes simplex can be identified employing serology [2, 22, 28, 29, 35].
- Hepatitis B, C and Syphilis: In addition, studies recommend testing for hepatitis B in unimmunized children and adolescent victims of sexual abuse [2, 27–30, 35]. Syphilis and Hepatitis B serologic testing must be repeated 6 weeks and 3 months later [29]. Testing for Hepatitis C should also be done [29].
- Pregnancy test: According to two clinical guidelines, the pregnancy test should be performed only in adolescent girls with delayed menstruation [2, 22].
- Cervical cytologic analysis: Carole et al., in their study, suggested that adolescent girls should have the first cervical cytologic analysis at the age of 21, except there are special conditions, such as HIV infection or immune suppression [25].

#### Prophylaxis for HIV and other STIs

A total of 12 studies suggested that children and adolescents who have been sexually abused, especially with oral, vaginal, or anal penetration, should receive early HIV Post-Exposure Prophylaxis (PEP) at the first 72 h after the last sexual abuse [2, 23–28, 30, 31, 39]. Sexually abused victims presenting later than 72 h are no longer indicated for antiviral prophylaxis [2, 29, 35]. HIV PEP should be started within 72 h of the sexual abuse and continued for 28 days [2, 22, 24, 26, 29]. According to two clinical guidelines, drug programs for PEP in adolscents are determined by local or national conventions [2, 22]. The latest WHO guidelines recommended two or three antiviral drugs for HIV PEP [2]. As mentioned in most studies, children and adolescents who have suffered sexual abuse may be infected with an STI [2, 23–28, 30, 31, 39]. Prophylaxis for bacterial and viral infection in sexually abused children and adolescence is needed, especially in settings where laboratory testing is not possible [2, 24]. According to the two Clinical Guidelines, drugs used as prophylaxis depend on the local protocol [2, 22]. The recommended antibiotics for prophylaxis in some studies consist of oral cefixime 400 mg or ceftriaxone, azithromycin 1 g, and Metronidazole 500 mg (for gonorrhea, chlamydia, and trichomoniasis respectively), followed by screening 2 weeks later [24, 27, 28]. Studies recommended that prophylaxis should be given within the first 72 h after the sexual abuse [22, 24, 25, 27, 28, 35].

#### Vaccinations

One study from the UK confirms that in countries with low childhood vaccination rates for tetanus and high prevalence of tetanus, prophylaxis against tetanus is recommended [24]. In addition, other studies reported that if there were mucosal or skin damages, a suitable tetanus toxoid (td) must be administered [22, 39]. Especially in unvaccinated and unprotected children, it may be necessary to administer anti-tetanus serum and a course of tetanus toxoid vaccine [22]. Some reviewed studies have suggested that unvaccinated children who have been sexually abused should be administered hepatitis B vaccination within 6 weeks of the last event [2, 22, 24, 29]. Vaccine first dose should be initiated at the time of examination, and then should be advised to complete three doses and a booster dose 12 months later [22, 24].

Furthermore, the national clinical practice guidelines suggested HPV vaccination is needed after child sexual abuse [29]. Two studies on child victims of sexual abuse also showed the effectiveness and importance of the HPV vaccine in post-rape care [36, 40]. HPV vaccination should be given to children between the ages of 9–14 years [2, 29].

#### Psychological intervention and follow up

Child and adolescent sexual abuse is a serious health concern with many short and long-term health consequences, negatively impacting the victim’s life [2, 24, 28–30, 32]. Some of these consequences based on the review of studies consist of: Post-Traumatic Stress Disorder (PTSD), low self-esteem, anxiety, decreased personal well-being, reduced school performance, loss of social competency, inappropriate sexual behavior, depression, substance abuse, and suicidal ideation [2, 21, 23, 27–30, 32–34, 37, 38]. The choice of behavioral or psychological intervention depends on victims’ ages and developmental stages and the behavioral and emotional problems [2, 37]. Based on the review of studies, psychological interventions used in children and adolescent victims of sexual abuse included:

- Psychological and social support and risk assessment of vulnerabilities, including domestic violence or self-harm, and practical support should be provided depending on victims’ needs [2, 24, 37],
- Trauma-Focused Cognitive Behavioral Therapy (TF-CBT) and follow-up for at least 6 months for sexually abused children and adolescents who had symptoms of Post-Traumatic Stress Disorder (PTSD) [2, 37]. Stress management may be useful for children and adolescents with PTSD [2].
- CBT and Interpersonal Psychotherapy (IPT) for children and adolescents with emotional disorders [2] and training care skills to parents and caregivers [2, 24].

## Discussion

Findings from the current systematic review provided a summary of the clinical interventions associated with providing care to child and adolescent victims of sexual abuse. These findings are mainly from studies conducted in middle- and high-income countries focusing on improving the care provided to victims.

In our study, both valid guidelines and articles on sexual abuse of children and adolescents were systematically examined. Through studies, we have found that providing timely and early care to children and adolescents who are victims of sexual abuse can reduce the short- and long-term effects of sexual abuse and thus improve their living conditions and adaptation.

In this study, by reviewing clinical guidelines and related studies on providing clinical care to children and adolescents victims of sexual abuse, it was found that the clinical services to victims of sexual abuse have undergone positive and efficient changes over time. Thus it is believed that in addition to other measures in the collection of evidence related to sexual abuse, newer tests such as DNA testing will increase the likelihood of obtaining evidence from aggressors even after the standard 72 h [26, 27]. Similarly, new studies reported that effective time in preventing unwanted pregnancies in female victims has increased from less than 72 h to 120 h after rape [2, 22, 25, 26, 35]. Likewise, whereasolder studies only focused on tetanus and hepatitis B vaccination, newer studies emphasize HPV vaccination in sexually abused children and adolescents [2, 29, 36, 40].

While the focus of this review was to provide evidence on clinical management and interventions to care for child and adolescent victims of sexual abuse, planning in health systems is needed to minimize harm to such victims. These plans include training health care approaches to health care providers, coordination mechanisms between the centers involved, monitoring and evaluation of systems, and the establishment of counseling and prevention services [18]. Resources, training, and an environment of supportive health systems are needed to provide trauma-informed care and critical early response. In fact, creating a supportive health environment enables health care providers to offer effective care and minimize victims’ feelings of shame, anxiety, and fear during interviews and examinations.

The strengths of this study include the extent of resources sought and the use of appropriate search methods and narrative synthesis to integrate the findings. The heterogeneity of the studies reviewed and the use of different quality assessment methods are limitations of our study.

## Conclusions

Suggestions for sexually abused children and adolescents include a set of evidence-based interventions. Many of these interventions are the same for sexually abused boys and girls. Also, these services for victims should be provided as soon as possible after sexual abuse, preferably less than 72 to 120 h. According to the results of this review, more studies have focused on prophylaxis against HIV and other STIs. Studies on vaccinating victims against HPV are limited, and future research in this area is recommended. The findings of the present review can be used by clinical development experts in child abuse and sexual violence against children and adolescents. Also, the recommendations of this study in 7 areas of care are in line with the principles derived from international human rights instruments such as the Convention on the Rights of the Child (CRC) and The Convention on the Elimination of All Forms of Discrimination against Women (CEDAW), which provide normative and protective standards to protect children and women from violence.

## Supplementary Information

**Additional file 1.**

## Data Availability

In this review all data are available from the referenced articles. The open access articles used for analysis during the current study are available from the corresponding author.
